# Air Particle Abrasion in Dentistry: An Overview of Effects on Dentin Adhesion and Bond Strength

**DOI:** 10.3390/dj13010016

**Published:** 2024-12-29

**Authors:** Andreea Kui, Smaranda Buduru, Anca Labuneț, Sorina Sava, Dalia Pop, Iris Bara, Marius Negucioiu

**Affiliations:** 1 Prosthetic Dentistry Discipline, Department 4—Prosthodontics and Dental Materials, Faculty of Dental Medicine, “Iuliu Hațieganu” University of Medicine and Pharmacy, 400012 Cluj-Napoca, Romania; gulie.andreea@umfcluj.ro (A.K.); dana.buduru@elearn.umfcluj.ro (S.B.); marius.negucioiu@umfcluj.ro (M.N.); 2 Dental Materials Discipline, Department 4—Prosthodontics and Dental Materials, Faculty of Dental Medicine, “Iuliu Hațieganu” University of Medicine and Pharmacy, 400012 Cluj-Napoca, Romania; savasorina@elearn.umfcluj.ro; 3 Cluj County Emergency Clinical Hospital, 400347 Cluj-Napoca, Romania; acteea.dali.pop@elearn.umfcluj.ro (D.P.);

**Keywords:** air particle abrasion, dentin adhesion, bioactive glass, hybrid layer, shear bond strength, dentistry

## Abstract

**Background/Objectives:** Air particle abrasion (APA) is a common surface preparation method in dentistry, particularly for improving bond strength to dentin. This review evaluates the influence of APA on dentin adhesion. **Methods**: A systematic literature search from 2018 to 2023 was conducted according to PRISMA-ScR guidelines. Articles investigating the effects of APA on dentin adhesion using different particle types, sizes and adhesive systems were included. Data extraction included particle size, air pressure, outcomes tested and failure modes. **Results**: Fourteen primary studies met the criteria. Bioactive glass showed higher bond strength and more cohesive failures than alumina. Alumina particles (50 μm) bonded effectively in etch-and-rinse adhesive systems but failed more often in self-etch systems. Silica-modified alumina and mixed abrasive systems showed improvements in bonding performance. Optimal APA parameters were identified as 50 μm particle size, 60 psi (4 bar) air pressure and 5 s exposure time. Longer exposure times provided no additional benefit. Self-etch systems showed reduced bond strength compared to etch-and-rinse systems. **Conclusions**: This review looks at how particle type, size and air pressure affect dentin adhesion. Bioactive glass is a superior material due to its bond strength and reduced cytotoxicity. The optimal APA parameters are 50 μm particle size, 60 psi and 5 s. Etch-and-rinse systems are recommended for optimal adhesion. Further research is required on APA protocols and long-term durability.

## 1. Introduction

The concept of air-abrasive technology, first introduced by R.B. Black in 1945 and later expanded upon by numerous researchers [1,2], represents a pivotal development in dental treatment methodologies. This technique uses compressed air to propel finely divided abrasive particles toward dental tissues to facilitate the removal of plaque. Air abrasion was originally intended to improve patient comfort during dental procedures [2] as an alternative to rotary dental burs.

Over time, air abrasion has found many applications in modern dentistry, including caries removal, periodontal surgery, orthodontics, enamel pretreatment, resin composite pretreatment, restorative dentistry and cavity preparation [3]. Each of these applications requires specific treatment protocols, as the abrasive material, air pressure, nozzle angle, nozzle–target distance and abrasion time must be carefully selected to achieve optimal results [4]. Despite its broad applicability, the lack of standardization of these key operating parameters has led to significant variability in clinical outcomes.

The most commonly used abrasives for air abrasion include alumina (Al_2_O_3_), sodium bicarbonate and glycine. However, recent studies have highlighted the growing interest in bioactive glass (BAG) as an alternative abrasive material. While sodium bicarbonate is primarily used for polishing procedures, aluminum oxide (alumina) has become a key material in the surface preparation of dental restorations, particularly in improving the micromechanical retention of restorative materials such as glass ceramics, oxide ceramics and resin composites [5,6]. The use of alumina aims to increase the surface roughness of dental substrates, thereby promoting stronger micromechanical interlock of adhesives. Recent research has suggested that bioactive glass (BAG) may offer comparable or superior performance to alumina, making it a promising alternative for surface conditioning in dental bonding [3,7,8].

The variability in the choice of abrasive material, particle size, air pressure and treatment protocols represents a significant gap in the literature, particularly with regard to the optimal combination of parameters required to achieve effective bonding to dentin. This discrepancy highlights the need for a systematic evaluation of the available evidence to provide clinicians with practical guidance for clinical decision-making. While experienced operators may select air abrasion parameters based on familiarity with the equipment, the lack of standardized protocols increases the risk of inconsistent results. Filling this gap could facilitate the development of evidence-based recommendations for air abrasion procedures, ultimately making them more predictable, efficient and cost-effective.

Several in vitro studies have investigated the effect of air particle abrasion (APA) on dentin surfaces with the goal of improving the bond strength between dentin and adhesive materials. Unlike surface preparation for indirect restorations such as ceramics and composites, APA on dentin is performed to increase surface area, improve surface energy, and promote better micromechanical interlock of adhesives. This focus on dentin bonding has significant implications for adhesive dentistry, as it directly affects the long-term clinical performance of adhesive restorations [8,9].

The objective of our research was to evaluate the effectiveness of APA in improving the bond strength between dental adhesives and dentin surfaces. This includes analyzing how different abrasion conditions (e.g., particle size, type, pressure and duration) and adhesive systems affect the quality of the bond. Therefore, in conducting this scoping review, we aimed to answer the following research questions: “Does air particle abrasion improve bond strength to dentin?” and “What settings of APA and adhesive system types are more favorable for adhesion to dentin?”.

## 2. Materials and Methods

This scoping literature review was conducted following the PRISMA-ScR guidelines (Preferred Reporting Items for Systematic reviews and Meta-Analyses extension for Scoping Reviews) guidelines [10]. The PRISMA-ScR checklist was applied to ensure methodological rigor and transparency throughout the review process. The research question was defined through the PICOT format (population, intervention, comparison, outcomes and time) [11]: P: dentin samples, either human or bovine; I: use of air particle abrasion; C: different particle dimensions, types and pressure; O: tensile or shear bond strength of various materials to dentin and alterations of dentin surface; and T: a contemporary overview of studies published between from June 2018 to June 2024. The decision to limit the search to a five-year period was based on the rapid evolution of adhesive bonding technologies and the introduction of novel materials, such as bioactive glass and modified airborne-particle abrasion techniques. This timeframe ensures that only the most relevant and up-to-date evidence is included, enhancing the clinical applicability of the findings.

This review used the Preferred Reporting Items for Systematic Reviews and Meta-Analyses (PRISMA) 2020 reporting guideline for both the study design and the abstract [11]. A detailed protocol was registered at the International Prospective Register of Systematic Reviews (PROSPERO number CRD42024607951).

### 2.1. Information Sources and Search Strategy

The search, conducted by two reviewers (AL and AK) from 1 September to 10 October 2024, utilized three bibliographic databases: Medline (PubMed), Scopus, and Embase. Three search concepts were established (Table 1), and relevant keywords and search terms, including MeSH terms, were developed for consistent use across all databases. Detailed search combinations for each database are provided in Table 2. Alongside database searches, a manual search was performed, and references from various studies were reviewed to identify additional relevant and eligible studies.

### 2.2. Eligibility Criteria

Inclusion criteria

1. In vitro studies—involving APA on dentin of the dental crown.
2. Articles focusing on APA involving different types of particles, various particle dimensions, and different air pressures, with or without water projection on dentin.
3. Studies published in English and completed between 2019–2024.

To ensure the integrity of the evidence synthesis, only primary, original research studies were included in the review. Previously included review articles have been excluded, and only data from primary sources were used for analysis and conclusions.

Exclusion criteria

1. Studies involving the use of APA on ceramics or composites to improve adhesion to tooth structures.
2. Studies performed below gingival level or in the root canal.
3. Studies on APA for bleaching or tooth cleaning purposes.
4. Studies on temporary teeth.
5. Studies that do not report any of the key outcomes of interest for the review.
6. Articles published in languages other than English, older than 5 years.
7. Other literature reviews, systematic reviews, or metanalyses.

### 2.3. Data Extraction and Method of Analysis

For data extraction, a standardized form was used and recorded on an Excel table (v.15.17—Microsoft, Redmond, WA, USA). The extracted information included the following: bibliographic details (authors, title, year of publication, journal), study design and methodology, sample size, outcomes (tensile or shear bond strength of various materials to dentin, alterations of dentin surface, etc.), key findings and conclusions.

To ensure consistency and minimize potential bias, two reviewers independently extracted data, resolving any discrepancies through discussion or by consulting a third reviewer. The reviewers also assessed the quality of the articles. A standardized template was created to extract key study information, such as study purpose, sample, groups, and relevant results aligned with the review’s objectives. Initial data extraction was performed by the two primary reviewers and subsequently reviewed for accuracy and completeness by two additional investigators (SB and SS).

## 3. Results

### 3.1. Data Collection

**Figure 1:**
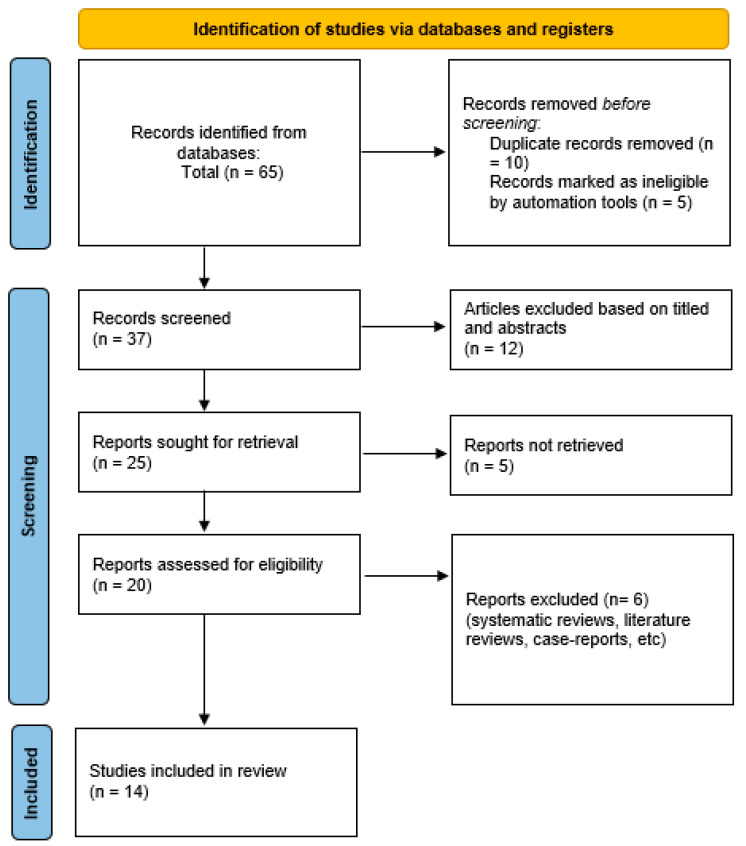
PRISMA flow diagram.

The search strategy (outlined in Table 1; Table 2) initially identified 65 articles. After removing duplicates and irrelevant records, 50 articles remained for screening. During the first phase, titles and abstracts were reviewed for relevance to the study question, narrowing the selection to 37 articles. Of these, 20 were further evaluated for eligibility, with any disagreements resolved through discussion and consultation with a third and fourth researcher. Ultimately, 14 publications were included in the review. The selection process and inclusion decisions are illustrated in Figure 1, the PRISMA flow diagram.

To ensure the integrity of the evidence included in this review, a systematic retraction check was performed for all included primary studies. The status of each study was verified using the Retraction Watch database, PubMed retraction notices, and the respective publishers’ journal websites. No retracted studies were identified among the included primary research articles.

### 3.2. Description of Included Studies

We identified 14 studies that investigate air particle abrasion effects on dentin adhesion. These studies analyze various methodologies and outcomes pertinent to the effectiveness and impact of air abrasion in dentistry. The characteristics and findings of these studies are organized in Table 3. Of these, 13 studies were in vitro studies, while 1 was a longitudinal study, and most of them were conducted across various countries, including the USA, Germany, India, and more. All studies were published between 2019 and 2024. The total number of studies that investigated bioactive effects on bond strength was four, while others assessed factors such as composite resin bond strength, enhancements in tensile strength, and the effectiveness of different air abrasion particles and techniques. Key findings include variability in bond strength effectiveness based on the type of abrasion particle used, with some significant improvements in bond strength and others showing no significant effects. No studies reported adverse effects directly associated with the dental or craniofacial aspects of air abrasion treatments.

### 3.3. Bond Strength and Failure Modes

The included studies demonstrate that airborne particle abrasion (APA) and bioactive glass play an important role in improving bond strength and failure mode at the adhesive–dentin interface. Studies using APA with alumina [12,17,19] demonstrated increased shear bond strength (SBS) and microtensile bond strength (μTBS), particularly when smaller particles (e.g., 27 µm) were used [19]. The presence of mixed and cohesive failure modes in APA-treated specimens suggests that the bond between the adhesive and dentin was stronger than the intrinsic strength of the dentin itself. On the other hand, studies using larger particles (e.g., 50 µm) reported higher adhesive failure rates, indicating a potential weakening of the adhesive–dentin interface [12,20]. This trend underscores the importance of optimizing particle size and air pressure to achieve durable bonds. The introduction of silica-modified alumina [19] was a promising alternative, with significantly fewer adhesive failures observed compared to conventional alumina.

Bioactive glass particles [16,18] have shown clear benefits in terms of long-term bond stability. Studies using bioactive glass showed higher bond strength and fewer adhesive failures, suggesting improved adhesion at the interface. For example, Spaguolo et al. reported that bioactive glass produced stronger bonds and had a greater proportion of cohesive and mixed failures, suggesting a more durable and stable adhesive interface [16]. In addition, Mavriqi et al. showed that water air particle abrasion (WAPA) with bioactive glass significantly increased microtensile bond strength (μTBS) and contributed to an impressive 15-year clinical survival rate that was significantly higher than that of untreated controls [25]. This finding highlights the potential of bioactive glass as a novel pre-treatment method to promote long-term bond strength. The presence of mixed and cohesive failures in bioactive glass-treated specimens reflect a strong bond at the adhesive–dentin interface, in contrast to the adhesive failures seen in untreated or aluminum oxide-treated specimens. Taken together, these findings support the clinical relevance of bioactive glass and WAPA protocols for improving long-term bond strength and reducing the risk of adhesive failure in bonded restorations.

### 3.4. Bias Assessment

Each included study was assessed against these criteria to identify sources of bias that could influence the results. Studies with strong methodological rigor, including clear randomization, blinding, adequate sample size, and appropriate statistical analyses, were rated as low risk of bias. Studies lacking key methodological details or failing to control confounders were rated as moderate to high risk of bias (Table 4).

## 4. Discussion

The use of air particle abrasion (APA) in restorative dentistry has been widely studied, particularly its effect on dentin adhesion and bond strength. This discussion addresses the key findings of the present scoping review, highlighting the influence of particle type, particle size, air pressure, treatment protocols and adhesive systems on bond performance.

### 4.1. Influence of Different Abrasive Particles

The type of particle used in APA is critical to its effectiveness. The most commonly studied materials include aluminum oxide (alumina), silica-modified aluminum oxide, bioactive glass, sodium bicarbonate and mixed or alternative particles.

Studies using alumina particles (typically 50 µm) have shown mixed results. While some studies report improvements in bond strength due to increased surface roughness and micromechanical interlocking, others have found no significant differences or even reductions in bond strength due to residual particle contamination on the dentin surface [12,14,17]. Alumina is often associated with adhesive failure, as seen in several studies where the adhesive–dentin bond was weaker than the dentin itself [19,20].

Silica-modified alumina, as tested by Levartovsky et al. [19], was a promising alternative. The silica-modified particle achieved higher bond strength values with a reduced incidence of bond failures compared to conventional alumina. This result is attributed to the improved surface chemical interaction of silica with adhesives.

Bioactive glass has also emerged as an important alternative. Studies by Spagnuolo et al. [16] and Mavriqi et al. [25] showed that bioactive glass particles not only improved bond strength but also increased the long-term stability of adhesive bonds. Importantly, bioactive glass did not interfere with the metabolic activity of pulp stem cells, unlike alumina particles [16]. In addition, bioactive glass produced higher cohesive failure rates, indicating a stronger bond at the adhesive–dentin interface compared to adhesive failures observed with alumina-based treatments [16,25].

Other studies have investigated the use of sodium bicarbonate and erythritol-based particles with mixed results. Sodium bicarbonate, typically used for air polishing rather than bond enhancement, has shown potential for smear layer removal, but its effect on bond strength is still controversial [17]. Erythritol-based particles have had a detrimental effect on bond strength, with studies reporting lower shear bond strength (SBS) values compared to controls [17].

### 4.2. Particle Size, Air Pressure and Duration

The performance of APA is also influenced by particle size, air pressure and duration of application. Variations in particle size, from 27 µm to 110 µm, have been extensively studied. Smaller particles (e.g., 27 µm) generally produce smoother surfaces and stronger bonds, as shown in studies by Melkumyan et al. [17] and Szerszen et al. [25]. Larger particles, e.g., 50 µm, produce rougher dentin surfaces but may leave particle debris that can compromise bond strength in self-etch systems [17].

With regard to air pressure, the optimal settings remain controversial. Studies have used pressures of 4 to 6 bar (60 to 80 psi). For example, Zhang et al. [14] found that 75 psi (5 bar) with 110 µm particles improved microtensile strength in sclerotic dentin, whereas Falcon Aguilar et al. [12] recommended 60 psi (4 bar) for optimal results. However, prolonged exposure to APA does not necessarily improve bond strength. A short, precise exposure (e.g., 5 s) has been reported to be sufficient to achieve effective results, as shown by Falcon Aguilar et al. [12].

### 4.3. Bonding Systems and Etching Techniques

The interaction of APA with different bonding agents and etching systems is a critical aspect of this review. Studies show that the bond strength varies between etch-and-rinse and self-etch systems. In most cases, APA improves bond strength when used in conjunction with an etch-and-rinse system, especially after phosphoric acid etching [12,19]. Conversely, in self-etch systems, residual particles of alumina or other abrasives can inhibit proper hybrid layer formation, resulting in reduced bond strength [20,21].

In one notable study, Ramos et al. [18] observed that G2 Bond Universal (G2B) performed better under etch-and-rinse conditions than under self-etch conditions. Bioactive glass demonstrated versatility, showing improved bond strength under both bonding approaches [18]. Studies with other adhesives, such as Scotchbond^®^, Adhese Universal^®^, and G-Premio Bond^®^, support the use of etch-and-rinse over self-etch modes, particularly for adhesives applied to air-abraded dentin [20].

### 4.4. Long-Term Bond Stability and Failure Modes

Failure mode analysis provides insight into the durability of the adhesive–dentin interface. Mixed and cohesive failures indicate stronger adhesive bonds, whereas adhesive failures indicate a weaker interface. Studies of bioactive glass, silica-modified alumina and alumina have shown clear differences in failure modes.

Bioactive glass was associated with a higher rate of cohesive failures, indicating that the adhesive–dentin bond was stronger than the dentin itself [16,25]. This finding highlights the durability and stability of bioactive glass bonds compared to other particles. In contrast, studies using alumina reported a higher frequency of adhesive failures, suggesting weaker bonds at the adhesive–dentin interface, especially when using self-etching systems [12,20].

Failure mode analysis is critical to understanding the long-term clinical applicability of different APA protocols. Cohesive failures are preferred in clinical dentistry, as they reflect a strong bond, whereas adhesive failures imply potential for bond failure over time.

The findings of our research, which highlight variable effects of APA on dentin bond strength, are in line with observations reported by Huang et al. (2019) [26]. Huang et al. demonstrated that air abrasion, particularly with aluminum oxide, can create a roughened surface that improves the adhesive bond in some cases but does not always enhance bond strength. This aligns with our results, where differences in particle size, air pressure and the use of bioactive glass influenced bond outcomes. The study by Almeida et al. (2024) [27] also supports this variability, reporting that 63.6% of studies found no influence of APA on bond strength, while 30.3% showed improvements and 6.1% reported reductions. This diversity in findings is consistent with our observation that bond strength is significantly influenced by experimental conditions, especially air pressure, particle type and adhesive system. Furthermore, Masse et al. (2021) [28] emphasize the importance of dentin surface roughness in achieving optimal bonding, noting that sandblasting enhances surface roughness but may increase failure rates under thermocycling. This comparative evidence further highlights that etch-and-rinse adhesives benefit more from air abrasion than self-etch systems, which is also reflected in our findings.

The observed inconsistencies in bond strength outcomes can be attributed to key differences in particle size, pressure and adhesive system across studies. For instance, while the study by Almeida et al. (2024) found that the majority of included studies reported no influence of APA on bond strength, it is important to note that many of these studies used larger particles (50 µm aluminum oxide), which, in our study, also led to lower bond strength [27]. On the other hand, studies employing smaller particles, such as 27 µm aluminum oxide or bioactive glass, reported more favorable outcomes, a pattern observed in our results as well. Additionally, the effect of aging protocols, such as thermocycling (10,000 to 50,000 cycles), plays a critical role in bond performance. Masse et al. (2021) emphasize that surface roughness induced by air abrasion increases hydrolytic degradation of the adhesive–dentin interface over time, which may explain why certain samples in our study exhibited adhesive failures after aging [28]. Furthermore, bioactive glass particles have been found to provide greater resistance to failure compared to aluminum oxide, as seen in both the work of Huang et al. (2019) and Almeida et al. (2024) [26,27]. This is attributed to the bioactive smear layer formed by bioactive glass, which may offer additional protection against water-induced degradation.

### 4.5. Clinical Relevance and Implications

This review highlights the clinical implications of using APA for dentin bonding. Clinicians are advised to carefully select the particle size, pressure and abrasive to optimize bond strength. Based on the findings of this review, the following recommendations are proposed: (1) bioactive glass is recommended due to its superior bond strength, minimal cytotoxicity and reduced bond failures; (2) alumina (50 µm) at 60 psi (4 bar) for 5 s is effective but should be followed by etch-and-rinse adhesive systems to avoid particle contamination; (3) etch-and-rinse bonding systems are preferred when using APA, especially in combination with phosphoric acid etching.

Failure mode analysis should be routinely performed in future studies to better understand bond durability and to guide clinical protocols.

### 4.6. Limitations of This Study

Although this review highlights key findings on APA and its effects on dentin bonding, several limitations should be noted. Firstly, the lack of standardization of APA parameters (e.g., particle size, pressure and duration) across studies limits direct comparisons. The predominance of in vitro studies raises concerns about the clinical applicability of these results, as in vitro conditions do not fully simulate the oral environment. In addition, the lack of long-term bond durability data prevents a comprehensive assessment of the clinical performance of APA.

While bioactive glass demonstrates promising effects on bond strength and long-term adhesion stability, it is important to acknowledge that only two studies in this review explicitly investigated its effects [19,28]. These studies provide initial but encouraging evidence, particularly in terms of stability after aging and survival rate improvements. Further research is warranted to draw more definitive conclusions regarding its clinical relevance.

Future research should focus on the standardization of APA protocols, long-term durability testing and the investigation of new bioactive abrasive particles. Clinical trials are needed to bridge the gap between in vitro results and in vivo performance. The inclusion of failure mode analysis as a standard measure in future studies will provide further insight into the long-term stability of adhesive–dentin bonds.

## 5. Conclusions

This review on air particle abrasion (APA) provides a complex view of the effectiveness of APA in the context of improving dental adhesion. Despite mixed results across studies, a common observation is the ability of APA to effectively remove the smear layer and achieve a thicker hybrid layer.

The type of abrasive particle has a significant effect on bond results. Bioactive glass has shown the most promising results, resulting in higher bond strength and a greater proportion of cohesive failures compared to other materials. Aluminum oxide remains effective, but the choice of bonding system (etch-and-rinse vs. self-etch) significantly affects its performance. Silica-modified alumina is also a viable alternative with favorable adhesion characteristics.

In addition, particle size, pressure, and time affect bond strength. Smaller particle sizes produce smoother surfaces with stronger bonds, while larger particles produce rougher surfaces with mixed results. Optimal air pressure is between 60 and 80 psi, while short exposure times (about 5 s) produce effective results without excessive dentin surface modification. Etch-and-rinse systems outperform self-etch when APA is used on dentin. This highlights the importance of adhesive selection, as APA may interfere with the formation of the hybrid layer.

Failure modes: The type of failure mode observed—adhesive, cohesive or mixed—reflects the quality of the adhesive–dentin interface. Bioactive glass shows a higher frequency of cohesive failures, indicating stronger adhesive bonds. Conversely, adhesive failures, particularly with self-etching adhesives, are more common with alumina-based treatments.

## Figures and Tables

**Table 1:** Search concepts.

Concept	Keywords & MeSH Terms
Air particle abrasion AND dentin	“Air” [Tw] AND particle [Tw] AND abrasion [Tw] AND “dentin” [Tw]
Sandblasting AND dentin	“sandblasting” [tw] AND “dentin” [tw]
APA AND dentin	“APA” AND “dentin”

**Table 2:** Search combinations per database.

Database	Search Terms and Combinations
PubMed Embase Scopus	“Air” [Tw] AND particle [Tw] AND abrasion [Tw] AND “dentin” [Tw] OR “sandblasting” [tw] AND “dentin” [tw] OR “APA” AND “dentin”

**Table 3:** Description of the included studies.

Reference	Population (P) Sample Size (Total/per Group and Group No.)	Intervention (I)	Comparison (C)	Outcomes (Intervention: I, Comparison: C)	Aging Protocol	Failure Mode
Falcon Aguilar et al. [12], 2024	Human third molars N = 72, n = 8/group	APA + PhoA (5s)	APA + PhyA (5s), or no APA	I: 73.25 ± 12.98 MPa; C: 61.85 ± 10.93 MPa	None	Adhesive and Mixed
Sinjari et al. [13], 2020	Bovine dentin (bars: 2 × 2 × 8 mm) N = 32 (Test: 16, Control: 16)	Sandblasting with 50 µm aluminum oxide particles + etching with 37% orthophosphoric acid	Only etching with 37% orthophosphoric acid	I: 84.30 ± 51.34 MPa (tensile stress), 18.54 ± 8.14 MPa (fracture stress); C: 35.07 ± 16.61 MPa (tensile stress), 88.19 ± 2.83 MPa (fracture stress)	None	Not explicitly mentioned
Zhang et al. [14], 2024	Premolars with noncarious cervical lesions (NCCLs) N = 32, nI = 16, nC = 16	Sandblasting with 110 µm aluminum oxide particles at 75 psi	No sandblasting, only resin composite	Microtensile bond strength (µTBS) I: 17.9 ± 0.69 MPa, C: 14.23 ± 0.44 MPa; Surface roughness (Ra) I: 1.01 ± 0.05 μm C: 0.16 ± 0.03	None applied	Not explicitly mentioned
Chaudhari et al. [15], 2024	Human premolars (flat occlusal surfaces) N = 60, 6 groups, n = 10/group	Acid etching (37% phosphoric acid), laser etching (Er:YAG), and air abrasion etching (27 µm alumina) for Giomer and G-aenial flo composites	No significant difference between techniques	Acid etching (Giomer: 11.8 ± 5.60 MPa, G-aenial: 11.7 ± 5.18 MPa) Laser etching (Giomer: 11.2 ± 2.87 MPa, G-aenial: 10.5 ± 5.90 MPa), Air abrasion (Giomer: 11.9 ± 4.74 MPa, G-aenial: 10.9 ± 2.89 MPa)	Thermocycling applied to simulate oral environment	Not explicitly mentioned
Spagnuolo et al. [16], 2021	Human molars (dentine specimens) N = 128, 4 groups, n = 32/group	Air-abrasion using Sylc Bioglass 45S5 (BAG), SELECTA (SEL), and Alumina (AL)	No air-abrasion (Smear layer: SML)	I: (BAG): 39.4 ± 4.7 MPa (baseline, T0), 35.1 ± 5.7 MPa (10 months); C: (SML): 40.2 ± 5.4 MPa (baseline, T0), 31.8 ± 4.9 MPa (10 months)	Aging in artificial saliva (10 months)	Mixed and Cohesive
Melkumyan et al. [17], 2021	Human molars (50 dentin specimens) N = 50, 5 groups, n = 10/group	Air-abrasion using alumina (50 µm, 27 µm), sodium bicarbonate (40 µm), or erythritol (14 µm)	No air-abrasion (control)	I: Alumina (50 µm): 28.53 ± 4.13 lb, Alumina (27 µm): 27.61 ± 3.44 lb, Sodium bicarbonate (40 µm): 25.92 ± 6.0 lb, Erythritol (14 μm): 23.05 ± 3.33 lb C: 28.35 ± 3.3 lb	None (one-day testing)	Not explicitly mentioned
Ramos et al. [18], 2021	Non-carious human third molars N = 70, 7 groups, n = 10/group	Airborne particle abrasion (AO29, AO53, SB, SBsoft, AT, BG)	Bur-cut dentin (control)	I: (BG): 67.1 ± 22.4 MPa (aged, E&R mode), C: (Control): 55.8 ± 23.4 MPa (aged, E&R mode)	Thermocycling (50,000 cycles)	Mixed (SE) and Cohesive (E&R)
Levartovsky et al. [19], 2023	Human molars (buccal and lingual dentin) N = 46, 2 groups, n = 23/group	Airborne particle abrasion (30 µm silica-coated alumina particles) + adhesive system (Clearfil SE or Scotchbond)	No air abrasion (control)	I: (Clearfil SE + SB): 22.58 ± 6.41 MPa; C: (Clearfil SE -SB): 21.33 ± 5.05 MPa; I: (Scotchbond + SB): 17.48 ± 6.75 MPa; C: (Scotchbond -SB): 14.33 ± 6.27 MPa	None applied (one-day testing	Mixed (52.2%) and Adhesive (58.3%)
Ouchi et al. [20], 2020	Bovine incisors (enamel and dentin surfaces) N = 600, 40 groups, n = 15/group	Aluminablasting with 50 µm aluminum oxide	No aluminablasting (control)	I: (Enamel, 24 h): 26.8–30.8 MPa; C: (Enamel, 24 h): 26.8–29.0 MPa; I: (Dentin): 23.1–28.8 MPa; C: (Dentin): 32.9–38.4 MPa	Thermocycling (30,000 cycles)	Adhesive (aluminablasting)/Mixed (without aluminablasting)
Kanzow et al. [21], 2020	Bovine enamel and dentin surfaces N = 400, 25 groups, n = 16/group	Sandblasting, silica coating, hydrofluoric acid etching	No contamination (control)	I: (Sandblasting, ER): 15.8 ± 4.3 MPa; C: (Control, ER): 25.7 ± 4.2 MPa	Thermocycling (10,000 cycles)	Not explicitly mentioned
Tepedino et al. [22], 2021	Human third molars (dentin surfaces) N = 60, 12 subgroups, n = 5/subgroup	Hydroabrasion (HA) with varying pressures (3 bar, 5 bar, 7 bar) + adhesive system application	Standard adhesive protocol without HA	I: (HA, 5 bar): 28.5 ± 7.9 MPa, C: (Control): 27.7 ± 10.8 MPa	Thermocycling (30,000 cycles)	Higher cohesive fractures in dentin
Szerszen et al. [23], 2022	Human molars (dentin surfaces N = 90, 3 groups, n = 30/group	Microabrasive blasting with Al_2_O_3_ (27 µm and 50 µm)	No microabrasion (control)	I: (27 µm): 6.25 ± 3.41 MPa; C: (Control): 2.89 ± 1.68 MPa	None applied (24 h water bath)	Not explicitly mentioned
Chauhan et al. [24], 2019	Human premolars (reduced crown height) N = 48, 4 groups, n = 12/group	Air abrasion with 50 µm alumina particles or Er:YAG laser irradiation	No surface treatment (control)	I: (Er:YAG): 4.88 ± 0.24 MPa; C: (Control): 3.97 ± 0.37 MPa	Thermocycling (5000 cycles)	Not explicitly mentioned
Mavriqi et al. [25], 2021	Human mandibular molars (N = 108; 2 groups: n = 40 (µTBS), n = 28(SEM))	WAPA with 50 µm alumina particles + 3-step etch-and-rinse adhesive system	Acid etching only	I: (WAPA): 63.9 ± 7.8 MPa, C: (Control): 51.7 ± 10.8 MPa	None (24 h water bath)	Not explicitly mentioned

APA: airborne particle abrasion; PhoA: phosphoric acid; PhyA: phytic acid; µTBS: microtensile bond strength; Er:YAG: Erbium-Doped Yttrium Aluminum Garnet Laser; SBS: shear bond strength; BAG: Bioglass 45S5; SEL: SELECTA (bioactive glass); AL: alumina; SML: smear layer; SB: sodium bicarbonate; SBsoft: soft sodium bicarbonate; AT: aluminum trihydroxide; BG: bioactive glass; SE: self-etch; E&R: etch-and-rinse; WAPA: waterborne airborne particle abrasion; HA: hydroabrasion.

**Table 4:** Bias assessment for the included studies.

Reference	Bias Assessment Method Used	Key Bias Criteria	Risk of Bias (Low, Moderate, High)	Justification
Falcon Aguilar et al. [12], 2024	No explicit bias assessment method reported	Randomization, blinding, outcome reporting	Moderate	Randomization was performed but lacked detailed reporting of baseline characteristics and blinding. Standardized protocols were used, but outcome assessor blinding was unclear.
Sinjari et al. [13], 2020	No explicit bias assessment method reported	Randomization, blinding, and outcome reporting	Moderate	Random allocation was used, but details on baseline characteristics and assessor blinding were unclear. Intervention protocols were well standardized. No thermocycling was conducted, which affects aging bias.
Zhang et al. [14], 2024	No explicit bias assessment method reported	Randomization, blinding, outcome reporting	Moderate	Randomization of teeth was mentioned, but no details were provided on randomization or blinding procedures for operators and assessors. Standardized interventions and objective measures were used.
Chaudhari et al. [15], 2024	No explicit bias assessment method reported	Randomization, blinding, outcome reporting	Moderate	Groups were clearly defined, but blinding of operators and outcome assessors was not mentioned. The study used standardized protocols and objective measures.
Spagnuolo et al. [16], 2021	No explicit bias assessment method reported	Randomization, blinding, outcome reporting	Moderate	The study used a large sample size with clear protocols but lacked details on blinding and assessor neutrality. Reporting was comprehensive, and the outcomes were objective.
Melkumyan et al. [17], 2021	No explicit bias assessment method reported	Randomization, blinding, and outcome reporting	Moderate	The study employed randomization into five groups but lacked details on blinding procedures. Standardized protocols and objective measures were used.
Ramos et al. [18], 2021	No explicit bias assessment method reported	Randomization, blinding, outcome reporting	Low	Randomization of dentin surfaces into seven groups was mentioned, but details on baseline characteristics and assessor blinding were not provided. Outcomes were objectively measured using µTBS testing.
Levartovsky et al. [19], 2023	Joanna Briggs Institute	Checklist, blinding, control group, clear protocol	Low	Randomization of teeth into groups was performed; objective measures (SBS, SEM) reduce detection bias.
Ouchi et al. [20], 2020	Joanna Briggs Institute Checklist	Randomization, blinding, outcome reporting	Low	Randomization of teeth into groups was performed; objective measures (SBS, SEM) reduce detection bias.
Kanzow et al. [21], 2020	No explicit bias assessment method reported	Randomization, blinding, outcome reporting	Moderate	The study randomized bovine teeth into groups, but blinding of operators and assessors was not mentioned. Shear bond strength (SBS) and failure analysis were objective and standardized.
Tepedino et al. [22], 2021	No explicit bias assessment method reported	Randomization, blinding, outcome reporting	Moderate	Teeth were randomized into three groups, and hydroabrasion conditions were standardized. However, blinding of operators and assessors was not mentioned. Objective measures (e.g., µTBS and SEM analysis) minimized bias.
Szerszen et al. [23], 2022	No explicit bias assessment method reported	Randomization, blinding, outcome reporting	Moderate	Random allocation of samples into groups was performed, but blinding of assessors and operators was not described. Shear bond strength (SBS) and SEM analyses were objective.
Chauhan et al. [24], 2019	No explicit bias assessment method reported	Randomization, blinding, outcome reporting	Low	Randomization of teeth into WAPA and non-WAPA groups was conducted; tensile bond strength was objectively measured using a universal testing machine.
Mavriqi et al. [25], 2021	Joanna Briggs Institute Checklist	Randomization, blinding, outcome reporting	Low	Randomization of teeth into WAPA and non-WAPA groups was conducted; objective measures (e.g., µTBS and SEM analysis) reduced bias. The JBI checklist categorized this study as high quality with a score of 7–10 out of 10, indicating low bias risk.

## Data Availability

Dataset available on request from the authors.
